# Effects of Proanthocyanidins on Growth Performance, Intestinal Inflammation and Barrier Function, and Bile Acid Metabolism-Related Genes in Weaned Piglets Challenged with Lipopolysaccharide

**DOI:** 10.3390/ani15131826

**Published:** 2025-06-20

**Authors:** Aiying Yu, Zhenjiang Wang, Sutian Wang, Weiguo Zhao, Lian Chen, Dan Wang, Zhiyi Li, Yuan Wang, Zhengfeng Fang, Sen Lin

**Affiliations:** 1 Key Laboratory for Animal Disease Resistance Nutrition of the Ministry of Education, Animal Nutrition Institute, Sichuan Agricultural University, Chengdu 611130, China; 2022214001@stu.sicau.edu.cn; 2 Key Laboratory of Urban Agriculture in South China, Sericultural & Agri-Food Research Institute, Guangdong Academy of Agricultural Sciences, Guangzhou 510640, China; wzj@gdaas.cn (Z.W.); chenlian@gdaas.cn (L.C.); wangdan@gdaas.cn (D.W.); lizhiyi2022@126.com (Z.L.); wangyuan18789@163.com (Y.W.); 3 State Key Laboratory of Swine and Poultry Breeding Industry, Guangdong Provincial Key Laboratory of Animal Breeding and Nutrition, Institute of Animal Science, Guangdong Academy of Agricultural Sciences, Guangzhou 510640, China; wstlyt@126.com; 4 Jiangsu Key Laboratory of Sericulture Biology and Biotechnology, School of Biotechnology, Jiangsu University of Science and Technology, Zhenjiang 212100, China; wgzsri@126.com

**Keywords:** proanthocyanidins, weaned piglets, growth performance, intestinal inflammation, barrier function, bile acid

## Abstract

This study aims to investigate the effects of dietary proanthocyanidins (PACs) on growth performance, intestinal inflammation and barrier function, and bile acid metabolism-related genes in weaned piglets challenged with lipopolysaccharide (LPS). A total of 18 21-day-old castrated piglets (7.16 ± 1.66 kg) were randomly assigned to three groups: (1) CON (a basal diet), (2) LPS (a basal diet + LPS), (3) LPS + PAC (a basal diet + LPS + 250 mg/kg PAC), with each group consisting of six replicates of 1 piglet per treatment. The study lasted for 21 days. On the 14th and 21st days of the experiment, piglets in the LPS and LPS + PAC groups received an intraperitoneal injection of 100 µg/kg body weight of LPS, while the piglets in the CON group received an injection of 0.9% normal saline solution. The LPS + PAC group exhibited a significantly higher average daily gain (ADG) than the LPS group (*p* < 0.05). LPS stimulation resulted in a decreased (*p* < 0.05) villus height of the jejunum and ileum and an increased number of goblet cells. These effects were alleviated (*p* < 0.05) in the LPS + PAC group. The LPS + PAC group decreased the level of TNF-α and D-lactate in serum and the gene expression of *IL-6* and *IL-1β* in the ileal tissue, compared with the LPS group, while increasing the gene expression of *Occludin* and *ZO-1* in the ileal tissue (*p* < 0.05). LPS stimulation down-regulated the expression of genes regulating bile acid synthesis and transport, including hepatic *CYP7A1* and ileum *ASBT*, whereas dietary PAC had no significant effect on the expression of these genes (*p* > 0.05). Nevertheless, supplementation with PAC significantly increased the expression levels of *GLP-2R*, *GCG*, and *TGR5* in the ileum of piglets (*p* < 0.05). Additionally, piglets in the LPS + PAC group exhibited a significant increase in the level of glucagon-like peptide 2 (GLP-2) compared with the LPS group (*p* < 0.05). PAC generally improves the ADG, intestinal morphology, and intestinal barrier function of piglets by activating TGR5 to stimulate the intestinal secretion of GLP-2.

## Simple Summary

The implications of growth performance, intestinal inflammation, barrier function, and bile acid metabolism-related genes in immune-stressed weaning piglets by proanthocyanidins (PACs) are observed. The results indicate that dietary supplementation with PAC was capable of improving the growth performance and intestinal health of LPS-challenged piglets. The beneficial effects of PAC towards the intestinal barrier function and inflammatory responses of piglets appeared to be attributed to the activation of TGR5 and subsequent secretion of GLP-2. These results are of significant scientific importance as they provide novel insights into the mechanisms by which PAC can mitigate the adverse effects of LPS-induced inflammation and improve intestinal health in weaned piglets.

## 1. Introduction

Weaned piglets encounter significant stress challenges in production, including environmental changes, alterations in feed, and the disappearance of maternal antibodies [1,2]. These stressors can lead to decreased growth performance and increased incidence of diarrhea [3]. Studies have shown that these results are associated with an increased expression of intestinal inflammatory genes, a decreased expression of tight junction proteins, impaired intestinal barrier function, and disrupted gut microbiota [4]. The addition of antibiotics to feed is a significant method for alleviating weaning stress and promoting the growth of weaned piglets in production settings [5]. Since the ban on feed antibiotics was implemented in China in 2020, intestinal health issues in weaned piglets have become increasingly prominent, resulting in substantial economic losses. Therefore, developing new antibiotic substitutes to safeguard the intestinal health of piglets is of great significance.

In recent years, proanthocyanidins (PACs), as natural plant extracts, have garnered considerable attention from numerous researchers due to their extensive advantages, including wide availability, environmental friendliness, and safety [6,7,8]. Recent studies have shown that dietary grape seed procyanidins (GSPs) resist intestinal oxidative stress by increasing intestinal microbial diversity and improving intestinal microbial balance [8]. Moreover, GSP regulates lipid metabolism in piglets by affecting the abundance of intestinal flora and the content of microbial propionic acid [9]. While the aforementioned studies have primarily focused on the perspective of intestinal flora metabolites, the underlying mechanisms by which proanthocyanidins regulate intestinal health remain unclear.

Previous studies have found that nutritional deficiency caused by weaning stress in piglets changes the composition of intestinal bile acids, which may affect intestinal barrier function by destroying intestinal tight junction proteins [4]. Therefore, bile acid homeostasis is a crucial factor influencing the intestinal health of weaned piglets, and the regulation of bile acid homeostasis is primarily mediated by bile acid receptors. The G-protein-coupled bile acid receptor 1 (GPBAR 1), also known as TGR5, is a member of the G-protein-coupled receptor (GPCR) superfamily and a key bile acid membrane receptor. Studies have shown that the activation of TGR5 can help alleviate the inflammatory response in macrophages [10] and regulate the barrier function of epithelial cells [11]. Accordingly, TGR5 is a significant target for modulating cell inflammation and barrier function. Moreover, studies have demonstrated that the incorporation of GSP into the diet enhances the expression of *GLP-1* in rat colon tissue [12]; GLP-1 is one of the markers of TGR5 activation [13,14]. Our previous study found that TGR5 activators alleviated the expression of inflammatory genes in the intestinal and liver tissues of piglets through GLP-2 [11]. Notably, GLP-2 is an important intestinal nutrition factor that has been widely confirmed to alleviate intestinal inflammation and protect intestinal mucosal barrier function [15,16]. Therefore, it was hypothesized that PAC activates the TGR5 receptor, thereby promoting the secretion of GLP-2 to repair intestinal health.

In summary, the objective of this study was to elucidate the mode of action by which PAC regulates intestinal inflammation and barrier function in weaned piglets. The result of this study may provide a new reference for the application of PAC as a new type of feed additive and clarify the potential value of bile acid receptor as a new target for intestinal health regulation in piglets.

## 2. Materials and Methods

All animal procedures were approved by the Institutional Animal Care and Use Committee of the Guangdong Laboratory Animals Monitoring Institute and complied with the current laws relating to animal protection (Ethics Approval Code: IACUC2024101).

### 2.1. Materials

PAC (purity ≥ 95%) was purchased from Tianjin Jianfeng Natural Products Research and Development Co., Ltd. (Tianjin, China). Lipopolysaccharide (*E. coli* O55:B5, L2880) was purchased from Sigma-Aldrich (St. Louis, MO, USA).

### 2.2. Animals, Diets, and Experimental Design

The feeding trial was conducted at the animal testing base of the Guangdong Laboratory Animals Monitoring Institute (Guangzhou, Guangdong, China). All weaned piglets tested negative for the major porcine enteric viruses, including porcine deltacoronavirus (PDCoV), porcine epidemic diarrhea virus (PEDV), transmissible gastroenteritis virus (TGEV), and rotavirus.

A total of eighteen 21-day-old castrated pigs (Duroc × Landrace, weaned at 21 d and fed the control diet for a 3 d adaptation period) with body weight (BW) of 7.16 ± 1.66 kg were randomly assigned to 3 treatments with 6 replicate pens: (1) control group (CON), fed a basal diet and injected with sterile saline; (2) lipopolysaccharides group (LPS), fed a basal diet and injected with LPS (100 µg/kg body weight); (3) lipopolysaccharides + proanthocyanidins group (LPS + PAC), fed a control diet supplemented with 250 mg/kg PAC and injected with LPS (100 µg/kg body weight). The study lasted for 21 days. The basal diet was formulated according to the NRC (2012), and the formula and nutritional composition are shown in Table 1.

All piglets were housed individually in an environmentally controlled animal facility. The room was maintained at 25–28 °C and approximately 55% relative humidity. The piglets were fed four times a day at 8:00, 12:00, 16:00, and 20:00, and had ad libitum access to food and water. According to previous research [17], on the morning of day 14 and day 21, the LPS and PAC groups were intraperitoneally injected with LPS. Meanwhile, the control group was intraperitoneally injected with an equal volume of sterile saline.

### 2.3. Sample Collection

At the end of the experiment, 2 h after the last injection with LPS, blood samples were obtained, and serum was separated by centrifugation at 3500 rpm for 10 min at 4 °C. Pigs were anesthetized by intramuscular injection of Zoletil (0.1 mL/kg body weight) and then slaughtered. The serum, liver tissue, ileum tissue, jejunum tissue, and colonic contents were collected and stored at −80 °C.

### 2.4. Piglet Growth Performance and Fecal Scores

The body weight of each piglet was recorded on days 0, 14, and 21 to calculate the average daily gain (ADG), and feed consumption was recorded for each replicate (pen) on days 14 and 21 to calculate the average daily feed intake (ADFI). The feed conversion ratio (FCR) was calculated as the ratio of ADFI to ADG. The diarrhea status of each piglet was observed daily during the feeding trial, and the fecal scoring criteria were as follows: grade 1, hard, dry, and friable feces; grade 2, normal feces; grade 3, pasty feces (mild diarrhea). Diarrhea index = total diarrhea score/(number of piglets × number of days).

### 2.5. Histomorphological Examination and Periodic Acid–Schiff (PAS) Staining of the Jejunum and Ileum Tissues

The tissue specimens were dehydrated step by step with ethanol and graded alcohol, cleared with a gradient of tert-butanol, and then embedded in paraffin wax. Consecutive sections (5 mm thick) were stained with hematoxylin–eosin (H&E) for histomorphological examination. The intestinal morphological structures were observed under 40× magnification using a light microscope. The villus height and crypt depth were measured in 10 randomly selected fields of view, and the mean values were calculated. Additionally, consecutive sections (5 mm thick) were stained with PAS to quantify the number of goblet cells under 40× magnification using a light microscope.

### 2.6. Serum Immune Indices and Immune Function

The levels of interleukin-6 (IL-6, CSB-E06786p), interleukin-1β (IL-1β, CSB-E06782), and tumor necrosis factor-α (TNF-α, CSB-E16980p-IS) were determined according to the manufacturer’s instructions. All ELISA kits were obtained from Cusabio Biotechnology Co., Ltd. (Wuhan, China). D-lactate (D-Lac, A019-3-1) and diamine oxidase (DAO, A088-3-1) were analyzed by the colorimetric method according to the protocols provided by the manufacturer (Nanjing Jiancheng Bioengineering Institute, Nanjing, China)).

### 2.7. Gene Expression

Total RNA was extracted from liver and ileum tissues using the SteadyPure Quick RNA Extraction Kit (AG21023, Accurate Biology, Changsha, China). The concentration of total RNA was determined using a spectrophotometer (NanoDrop 2000, Thermo Scientific, Wilmington, DE, USA), and 2 µg of total RNA was used for cDNA synthesis with the reverse transcription master mix (AG11728, Accurate Biology, Changsha, China) according to the manufacturer’s instructions. Relevant gene sequences were obtained from GenBank, and primers were designed using the National Center for Biotechnology Information (NCBI) Primer-BLAST tool (2.5.0) and synthesized by Sangon Bioengineering Co., Ltd. (Shanghai, China). The primer sequences used in the qPCR amplification reaction are shown in Table 2. The PCR amplification procedure was performed using a Roche fluorescence quantitative PCR instrument (Bio-Rad Laboratories, Inc., Hercules, CA, USA) according to the kit instructions (Accurate Biology, Changsha, China). The relative level of mRNA expression was calculated using the 2^−ΔΔCt^ method after normalization with β-actin as the housekeeping gene.

### 2.8. Data Processing and Statistics Analysis

All data were analyzed using a one-way ANOVA procedure in RStudio (4.3.3) and are expressed as the means ± SEM. Multiple comparisons were performed using the Least Significant Difference (LSD) test. Statistical significance was indicated by *p* < 0.05.

## 3. Results

### 3.1. Growth Performance

As shown in Table 3, PAC did not influence the growth performance of the piglets from D1 to D14 (*p* > 0.05). The piglets in the LPS + PAC group had an increased average daily gain (ADG) during the second stage (D14–D21) compared with the LPS group (*p* < 0.05).

### 3.2. Intestinal Morphology

**Figure 1:**
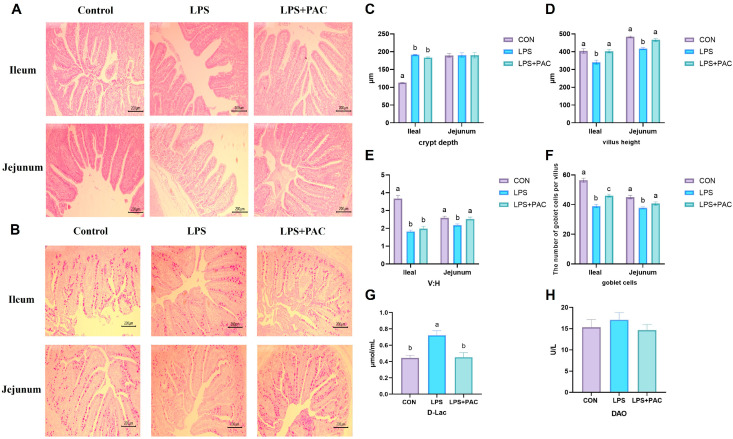
Effects of supplementing PAC on intestinal morphology and barrier function of weaned piglets. (**A**) Ileum and jejunum tissue H&E staining (40× magnification). (**B**) Ileum and jejunum tissue PAS staining (40× magnification). (**C**) Crypt depth. (**D**) Villus height. (**E**) V:H. (**F**) Number of goblet cells. (**G**) D-Lac content. (**H**) DAO content. The data are represented as means ± SEM, *n* = 5. Different letters indicate significant differences (*p* < 0.05).

The effects of PAC on the morphology and structure of the ileum and jejunum in immune-stressed piglets are shown in Figure 1A. Compared with piglets in the LPS group, piglets in the PAC group had a higher crypt depth in the jejunum (*p* < 0.05) (Figure 1B). Compared with those in the CON group, the villus height and V:C ratio in the jejunum and ileum of piglets in the LPS group were significantly lower (*p* < 0.05) (Figure 1C,D).

### 3.3. Intestinal Permeability

As shown in Figure 1C, the number of goblet cells in the PAC group was significantly greater than that in the LPS group (*p* < 0.05) (Figure 1C). As shown in Figure 1F,G, there was no significant difference in serum DAO content among the groups, and LPS stimulation significantly increased serum D-Lac acid levels in piglets. Compared with the LPS group, the serum D-Lac level of piglets in the LPS + PAC group was significantly decreased (*p* < 0.01).

### 3.4. Cytokine Content

**Figure 2:**
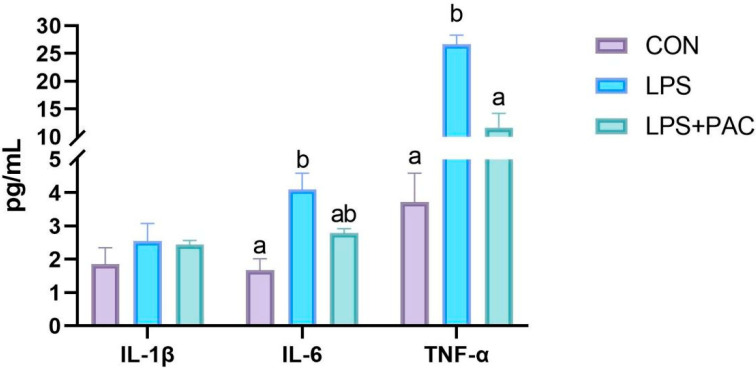
The serum inflammatory cytokine levels of IL-1β, IL-6, and TNF-α. The data are represented as means ± SEM, *n* = 5. Different letters indicate significant differences (*p* < 0.05).

The results are shown in Figure 2. Compared with the CON group, the LPS group showed a significant increase in the levels of IL-6 and TNF-α in serum (*p* < 0.05). The levels of TNF-α in serum were significantly decreased after PAC treatment (*p* < 0.05), while there was no significant difference in IL-6 levels compared with the LPS group (*p* > 0.05).

### 3.5. Gene Expression of Ileum Inflammatory Factors and Tight Junction Proteins

**Figure 3:**
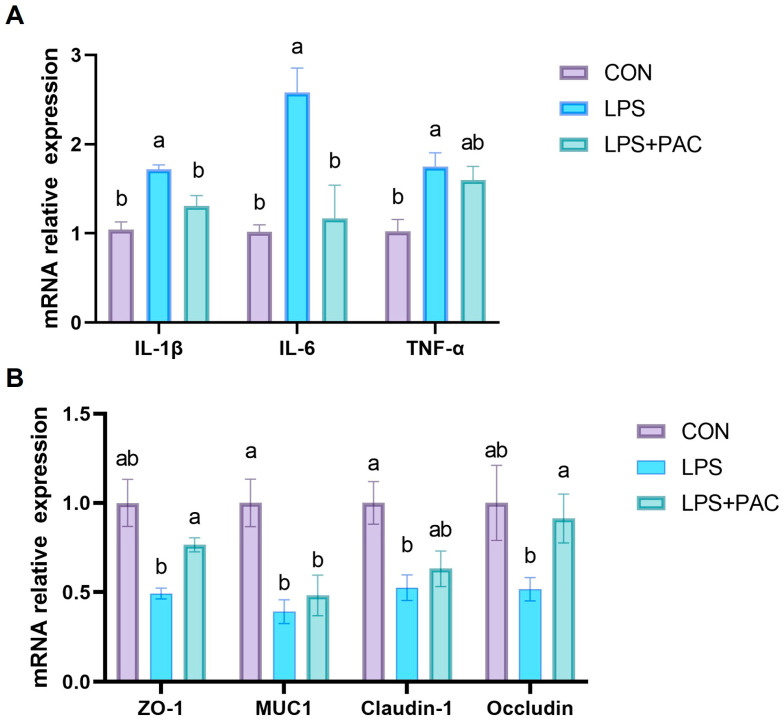
The mRNA expression levels of ileum inflammatory factors (**A**) and tight junction protein (**B**). The data are represented as means ± SEM, *n* = 5. Different letters indicate significant differences (*p* < 0.05).

The effects of PAC on the intestinal inflammatory cytokine factors genes of piglets are shown in Figure 3A. LPS injection significantly increased the gene expression of *IL-1β*, *IL-6*, and *TNF-α* in the ileum of piglets, while PAC supplementation significantly decreased the gene expression of *IL-1β* and *IL-6* in the ileum of piglets (*p* < 0.05).

The effects of PAC on the expression of tight junction proteins of piglets are shown in Figure 3B. The results show that the intraperitoneal injection of LPS significantly reduced the expression levels of *MUC1* and *Claudin-1* in the ileum of piglets (*p* < 0.05). The expression levels of tight junction proteins *Occludin* and *ZO-1* in the ileum of piglets supplemented with PAC were significantly higher (*p* < 0.05) than those in the LPS group.

### 3.6. Hematological and Biochemical Indices

The effects of PAC on blood physiological and biochemical indices of weaned piglets are shown in Table 4. After LPS injection, the number of red blood cells, hemoglobin content, hematocrit, and eosinophils in the blood of piglets was significantly decreased (*p* < 0.05). Compared with the LPS group, dietary PAC supplementation significantly increased hematocrit, mean corpuscular volume, mean corpuscular hemoglobin content, standard deviation of red blood cell distribution width, and aspartate aminotransferase in weaned piglets (*p* < 0.05). Compared with the CON group, the levels of eosinophils and total bilirubin in the LPS + PAC group were significantly decreased (*p* < 0.05).

### 3.7. Gene Expression of Liver and Ileum of Bile Acid Synthesis and Transport-Related Proteins

**Figure 4:**
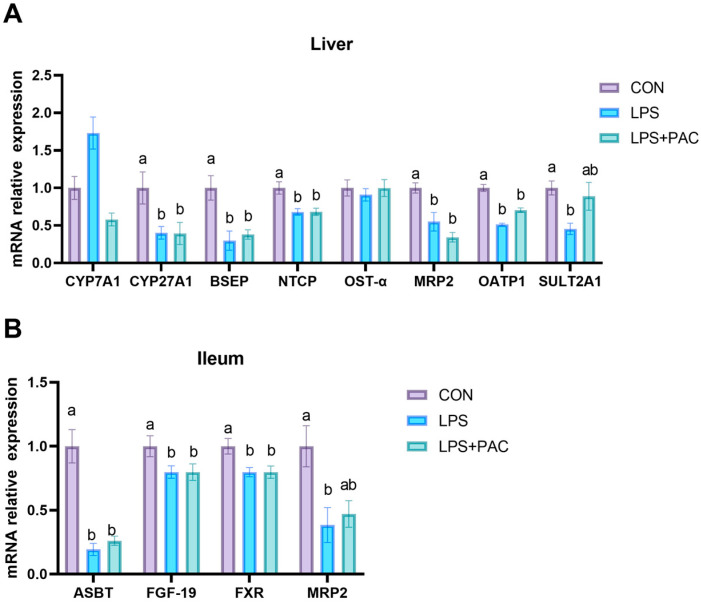
The relative mRNA expression of genes related to bile acid metabolism liver (**A**) and the ileum (**B**) of piglets. The data are represented as means ± SEM, *n* = 5. Different letters indicate significant differences (*p* < 0.05).

The expression of bile acid metabolism-related genes in liver tissue is shown in Figure 4A. Compared with the CON group, LPS stimulation reduced the expression of *BSEP*, *CYP27A1*, *NTCP*, *MRP2*, *OATP1*, *OSTα*, and *SULT2A1* in the liver tissue of piglets (*p* < 0.05). The expression of bile acid metabolism-related genes in the ileum tissue is shown in Figure 4B. Compared with the CON group, the expression levels of *ASBT*, *FGF-19*, and *FXR* in the ileum tissue of the LPS and LPS + PAC groups were significantly decreased, and the expression of *MRP2* in the LPS group was significantly decreased (*p* < 0.05).

### 3.8. Gene Expression of GLP-2 Related Genes in Ileum of Piglets

**Figure 5:**
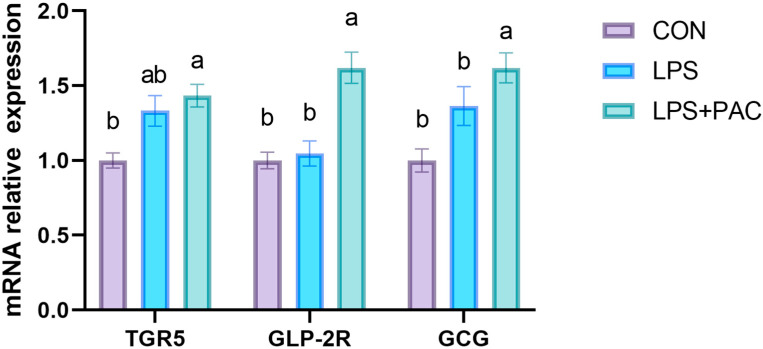
Expression of GLP-2 related genes in ileum of piglets. The data are represented as means ± SEM, *n* = 5. Different letters indicate significant differences (*p* < 0.05).

The effects of PAC on the expression of GLP-2-related genes in the ileum of weaned piglets are shown in Figure 5. Compared with the CON group, dietary PAC supplementation significantly increased the expression levels of *TGR5*, *GLP-2R*, and *GCG* in the ileum tissue (*p* < 0.05).

### 3.9. The Content of Serum GLP-1 and GLP-2 of Piglets

**Figure 6:**
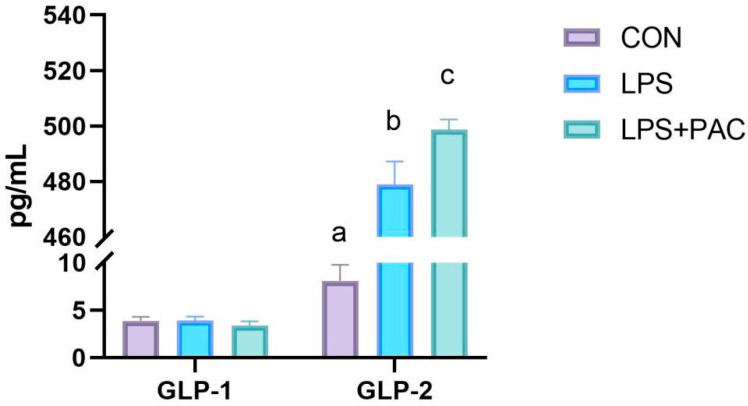
The content of serum GLP-1 and GLP-2 of piglets. The data are represented as means ± SEM, *n* = 5. Different letters indicate significant differences (*p* < 0.05).

As shown in Figure 6, there was no significant difference in serum GLP-1 levels among the three groups of piglets, and all were at a lower level (*p* > 0.05). Compared with the control group, the serum GLP-2 levels of piglets in the LPS and LPS + PAC groups were significantly increased (*p* < 0.01), and the serum GLP-2 level of piglets in the PAC group was significantly higher than that in the LPS group (*p* < 0.05).

## 4. Discussion

PAC holds significant potential as an alternative to antibiotics in animal husbandry, exhibiting notable applications in enhancing animal production performance and exerting antioxidant effects. Previous studies have shown that a diet supplemented with 250 mg/kg PAC improved the growth performance and reduced the incidence of diarrhea in weaned piglets [18]. Additionally, PAC supplementation at doses ranging from 30 to 120 mg/kg decreased FCR in growing pigs [19]. In the current experiment, no significant difference in growth performance was observed in piglets supplemented with 250 mg/kg PAC during the initial stage (D0-D14), which is consistent with the findings of Chedea et al. [20]. However, after the intraperitoneal injection of LPS, the ADG of piglets fed the PAC-supplemented diet was significantly improved compared to the LPS group during the second stage (D15-D21). Differences in ADG began to emerge in the second stage, and this was likely due to the LPS injection. The LPS injection triggered an immune response in the control-group piglets, which may have more severely affected nutrient metabolism and utilization. However, the PAC-group piglets, having consumed PAC and thus acquired stronger immunity, were relatively less affected by the LPS injection. On the other hand, LPS stimulation severely impaired the intestinal structure and function in piglets fed the basal diet. However, PAC supplementation safeguarded the intestinal barrier and attenuated the inflammatory response elicited by LPS, ultimately resulting in a better growth trend.

The intestine is the largest digestive and immune organ in animals, serving as the key barrier for nutrient acquisition and resistance to pathogen invasion. LPS causes intestinal villus atrophy, shallow crypts, inflammatory cell infiltration, and other phenomena, which are consistent with the results of this experiment [21]. This study demonstrated that PAC mitigates the morphological and structural damage to the ileum and jejunum of piglets following LPS stimulation. Wu et al. added four levels of PAC to broiler feed and found that it could alleviate the intestinal morphological damage caused by LPS stimulation, which was consistent with the results of this experiment [22]. When the body is subjected to immune stress, the intestinal mucosa is damaged and intestinal permeability increases, resulting in D-Lac entering the bloodstream via the cell bypass and the release of DAO into the bloodstream [23]. In this experiment, LPS challenge resulted in a significant increase in serum D-Lac content in piglets, and the addition of PAC to the feed alleviated this trend. These findings indicated that LPS challenge increased intestinal permeability in piglets, while PAC supplementation improved intestinal barrier function. It is speculated that PAC has the potential to enhance the integrity of the intestinal epithelial barrier caused by LPS stimulation. Goblet cells, as the primary mucin-secreting cells in the intestinal epithelium, are responsible for synthesizing and secreting mucins to form a protective mucus layer that covers the intestinal mucosa, thereby preventing bacterial invasion of the intestinal epithelium [24]. LPS also resulted in a decrease in the number of goblet cells in the ileum and jejunum of piglets, which is consistent with the results of this study [25]. The results of this experiment showed that supplementation with PAC alleviated the decrease in the number of goblet cells caused by LPS stimulation. As a peripheral cytoplasmic protein, ZO-1 regulates the assembly of tight junction proteins by anchoring the transmembrane proteins [26] Occludin and Claudins in the intracellular skeleton, which plays an important role in maintaining intestinal epithelial permeability [27]. The results of this study showed that LPS treatment significantly reduced the expression of *MUC1*, *ZO-1*, *Occludin*, and *Claudin-1* in the ileum mucosa of weaned piglets. The addition of PAC to the diet significantly reversed the down-regulation of *ZO-1* and *Occludin* expression induced by LPS. This finding is consistent with previous studies showing that the addition of lychee-derived PAC to the diet can mitigate the reduction in intestinal tight junction protein expression in DSS-induced colitis mice [28]. Consequently, PAC has the potential to restore the intestinal mucosal mucus layer and mitigate intestinal damage induced by LPS stimulation.

The integrity of the intestinal epithelium is closely related to the intestinal immune response [29]. Intestinal barrier immune dysfunction results in the overproduction of pro-inflammatory cytokines, which in turn may induce intestinal mucosal inflammation [30]. Previous research has demonstrated that PAC exerts a direct regulatory influence on immune cell signal transduction, pathogen sequestration, and the integrity of the intestinal mucosal barrier [31]. Furthermore, Hao et al. [32] and Fang et al. [8] demonstrated that the addition of procyanidins at concentrations of up to 1.5% and 1% in piglet diets significantly enhanced the activities of glutathione peroxidase and superoxide dismutase in the serum and liver, respectively. In this study, we investigated the effects of PAC on intestinal inflammation after injection with LPS. The results showed that feeding PAC reduces the level of TNF-α in serum and the gene expression of *IL-6* and *IL-1β* in the ileum tissue. Similar studies have also pointed out that PAC has a significant protective effect on dextran sulfate sodium (DSS)-induced colitis mice, trinitrobenzene sulfonic acid (TNBS)-induced colitis mice, and acetic acid-induced colitis mice, which is consistent with the results of this study [33,34]. Thus, our findings demonstrate that PAC significantly ameliorates LPS-induced intestinal inflammation in piglets.

Studies have demonstrated that LPS exerts an inhibitory effect on the expression of genes associated with bile acid synthesis and bile acid transport [35]. The results of this study showed that the low expression of *CYP27A1* in the liver of piglets after LPS treatment also reflected the inhibition of bile acid synthesis in the liver. Secondly, the low expression of *BSEP*, *ABCC2*, and *SLC01A2* in the liver reflects the damage to liver transport function, leading to the inability of bile acids to be effectively excreted. In addition, the low expression levels of *OSTβ* and *NTCP* in the liver and FXR and ASBT in the ileum also reflected the abnormal enterohepatic circulation of bile acids in piglets, resulting in the decreased reabsorption of bile acids and a decreased overall level. However, dietary supplementation with PAC did not mitigate the disruption of bile acid metabolism caused by LPS. GSPE had no significant effect on the expression of *CYP27A1* in rats, which was similar to the results of this study [36].

Previous studies have found that the treatment of intestinal bile acids may promote the secretion of GLP-1 and GLP-2 by activating TGR5 [37,38]. In this experiment, LPS-treated piglets exhibited increased serum GLP-2 concentration, and PAC further increased serum GLP-2 levels. In a study of piglets with short bowel syndrome, Lin et al. found that intestinal resection increased serum GLP-2 levels and enhanced intestinal weight, length, villus height, and crypt cell proliferation in the remaining intestine [11]. Therefore, we speculate that following intestinal damage in piglets, the body may elicit an adaptive response, which in turn stimulates a significant increase in GLP-2 secretion. However, there was no significant change in GLP-1 levels among the three groups. This may be due to its short half-life in the blood and the cleavage of DPP-4 and neutral endopeptidase in the receptor body, which inactivates GLP-1 [39]. In addition, the expression of TGR5, Proglucagon, and GLP-2R in the PAC group was significantly increased in the terminal ileum tissue. GLP-2 has a variety of physiological effects on the intestine. By activating the GLP-2 receptor, it stimulates the proliferation of intestinal crypt cells, inhibits apoptosis, maintains tight junction proteins in intestinal epithelial cells, and promotes the repair of intestinal mucosa after injury [40,41]. Therefore, PAC activates TGR5 to increase GLP-2 levels, thereby repairing intestinal health.

## 5. Conclusions

In conclusion, LPS challenge led to disrupted intestinal morphology and barrier function and up-regulated inflammatory genes in piglets, whereas dietary supplementation with PAC was capable of improving the intestinal morphology and barrier function of LPS-challenged piglets. The beneficial effects of PAC towards intestinal barrier function and inflammatory responses of piglets appeared to be attributed to the activation of TGR5 and subsequent secretion of GLP-2.

## Figures and Tables

**Table 1:** Ingredients and nutrient levels of the basal diet (%, as-fed basis).

Ingredients	Content %	Nutrition Level ^3^	Content %
Corn	28.85	Digestible energy, MJ/kg	14.83
Expanded corn	10.00	Metabolizable energy, MJ/kg	14.23
Extruded soybean	14.00	Net energy, MJ/kg	10.25
Fermented soybean meal	11.50	Crude protein %	21.11
Soybean powder	7.00	Calcium Ca %	0.90
Fishmeal	3.00	Total phosphorus %	0.63
Whey powder	15.00	SID lysine %	1.40
Whey protein concentrate	1.00	SID methionine %	0.40
Soyabean oil	1.00	SID threonine %	0.82
Sucrose	3.00	SID tryptophan %	0.23
Calcium citrate	1.85	SID isoleucine %	0.72
Dicalcium phosphate	0.60	SID methionine + cystine %	0.75
Sodium chloride	0.25		
L-lysine	0.55		
DL-methionine	0.15		
L-threonine	0.20		
L-tryptophan	0.05		
L- valine	0.10		
Choline chloride	0.20		
Vitamin premix ^1^	0.40		
Mineral premix ^2^	1.30		

^1^ The vitamin premix provided the following per kg of the diet: Vitamin A, 12000 IU; Vitamin B_1_, 5 mg; Vitamin B_2_, 6 mg; Niacin, 20 mg; Pantothenic acid, 20 mg; Vitamin B_6_, 3 mg; Biotin, 360 μg; Folic acid, 2 mg; Vitamin B_12_, 30 μg; Vitamin D_3_, 5000 IU; Vitamin E, 50 mg; Vitamin K_3_, 5 mg. ^2^ The mineral premix provided the following per kg of the diet: Fe (FeSO_4_·H_2_O), 100 mg; Zn (ZnSO_4_·H_2_O), 100 mg; Cu (CuSO_4_·5H_2_O), 6 mg; Mn (MnSO_4_·H_2_O), 4 mg; Se (Na_2_SeO_3_), 0.5 mg; I (KI), 0.2 mg. ^3^ Nutritional levels are calculated.

**Table 2:** List of primer sequences used for quantitative real-time PCR.

Gene	Primer Sequence (5′-3′)		Product Size (bp)
*β-actin*	FW	AGAGCAAGAGAGGCATCCTG	111
	RW	CACGCAGCTCGTTGTAGAAG	
*IL-6*	FW	TGTC GAGGCTGTGCAGATTA	102
	RW	GCATTTGTGGTGGGGTTAGG	
*IL-1β*	FW	AGACCCCAAAAGATACCCAAAGAGG	129
	RW	TCTGCTTGAGAGGTGCTGATGTAC	
*TNF-α*	FW	GGCCCAAGGACTCAGATCAT	105
	RW	GCATACCC ACTCTGCCATTG	
*ZO-1*	FW	TCCTGAGTTTGATAGTGGCGTTGAC	148
	RW	CACGGTGTGACCATCCTCATCTTC	
*Occludin*	FW	TTACTCCTACGCTGGTGACAACATTG	93
	RW	TGGATCTGCCCGGTGCTCTG	
*Muc1*	FW	GTGCCGCTGCCCACAACCTG	141
	RW	AGCCGGGTACCCCAGACCCA	
*Claudin-1*	FW	AGAAGATGCGGATGGCTGTC	193
	RW	CCCAGAAGGCAGAGAGAAGC	
*BSEP*	FW	GCCTGACCACGAGCATCT	60
	RW	AGGTCAGTTTCCAACCCTGAT	
*ABST*	FW	ACTTTCGGAAACCTAAGGGACT	88
	RW	AAGAGCTTGCCCAGTGCAAAG	
*NTCP*	FW	GTGGTACCCGAGAGCAACTT	132
	RW	CTTTACGTGCCCCAGGAACT	
*CYP7A1*	FW	GAAAGAGAGACCACATCTCGG	123
	RW	GAATGGTGTTGGCTTGCGAT	
*CYP27A1*	FW	GAACTCACTCTACGCCACCTTCC	85
	RW	GGTATTCCAGCCATCCAGGTATCG	
*OSTα*	FW	GGGTGAACTCTGAGATAGGAAC	73
	RW	CTGGGTCTTTCCTTCTGGCT	
*MRP2*	FW	TGCAAGTACGGACCAGTGTC	83
	RW	AACGGTGTACTGCTTCCTGG	
*OATP1A2*	FW	TGGCTGTGGTGGTCGTGATAAG	82
	RW	TCCGATGGCAGCGTCTTTGG	
*SULT2A1*	FW	CCATGCGAGACAAGGAGAAC	155
	RW	CATGACCTGGAAGGAGCTGT	
*FXR*	FW	TTTGTGTCGTTTGCGGAGAG	128
	RW	GTTGCCCCCATTTTTACACTTG	
*FGF-19*	FW	AAGATGCAAGGGCAGACTCA	101
	RW	AGATGGTGTTTCTTGGACCAGT	
*TGR5*	FW	CCATGCACCCCTGTTGCT	66
	RW	GGTGCTGTTGGGTGTCATCTT	
*GCG*	FW	CTCGATAATCTTGCCACCCGA	115
	RW	CTGGCAGGTGATGTTGTGAAC	
*GLP-2R*	FW	CCCTGCTGTTTCTGGTTTCC	195
	RW	GGCAGGGAACAGAAACGTTT	

**Table 3:** Effects of PAC on growth performance of weaned piglets.

Items	Treatments			*p*-Value
	CON	LPS	LPS + PAC	
BW (kg)				
1 d	7.09 ± 0.15	7.12 ± 0.14	7.14 ± 0.15	0.941
14 d	11.93 ± 0.29	11.47 ± 0.27	11.67 ± 0.46	0.815
21 d	16.52 ± 0.30	14.28 ± 0.27	15.33 ± 0.46	0.247
1–14 d				
ADG (kg/d)	0.35 ± 0.01	0.31 ± 0.01	0.35 ± 0.01	0.624
ADFI (kg/d)	0.51 ± 0.01	0.51 ± 0.01	0.52 ± 0.02	0.954
FCR	1.49 ± 0.01	1.65 ± 0.03	1.53 ± 0.03	0.165
Diarrhea index	0.48 ± 0.03	0.52 ± 0.04	0.27 ± 0.03	0.761
15–21d				
ADG (kg/d)	0.66 ± 0.02 ^a^	0.40 ± 0.02 ^c^	0.46 ± 0.02 ^b^	0.005
ADFI (kg/d)	0.97 ± 0.02 ^a^	0.73 ± 0.02 ^b^	0.81 ± 0.03 ^ab^	0.030
FCR	1.48 ± 0.01 ^b^	1.86 ± 0.03 ^a^	1.82 ± 0.04 ^a^	0.004
Diarrhea index	0.14 ± 0.03 ^b^	0.64 ± 0.05 ^a^	0.23 ± 0.05 ^ab^	0.015

Data are expressed as means ± SEM, *n* = 5. The superscript with no letter in common means that the difference is significant (*p* < 0.05). CON, control; LPS, lipopolysaccharide; LPS + PAC, lipopolysaccharide + proanthocyanidin. BW = body weight, ADG = average daily gain, ADFI = average daily feed intake, FCR = feed conversion ratio.

**Table 4:** Effects of PAC on serum routine blood test of weaned piglets.

Items	Treatments			*p*-Value
	CON	LPS	LPS + PAC	
Hematological indices				
WBC (10^9^/L)	13.70 ± 0.38	14.42 ± 0.63	16.69 ± 0.82	0.43
RBC (10^12^/L)	6.48 ± 0.05 ^a^	6.03 ± 0.04 ^b^	6.03 ± 0.03 ^b^	0.02
Hemoglobin (g/L)	109.50 ± 0.52 ^a^	98.00 ± 0.66 ^b^	105.40 ± 0.95 ^b^	<0.01
Hematocrit (%)	38.25 ± 0.22 ^a^	34.30 ± 0.25 ^b^	37.28 ± 0.27 ^a^	<0.01
MCV (fL)	59.08 ± 0.41 ^ab^	56.93 ± 0.26 ^b^	61.86 ± 0.55 ^a^	0.02
MCH (pg)	16.95 ± 0.15 ^ab^	16.28 ± 0.09 ^b^	17.46 ± 0.15 ^a^	0.09
MCHC (g/L)	286.50 ± 0.77	285.67 ± 0.68	282.80 ± 0.94	0.46
Platelets (10^9^/L)	693.00 ± 23.16	721.67 ± 9.98	834.60 ± 17.81	0.13
RDW-SD (fL)	37.93 ± 0.22 ^b^	39.10 ± 0.34 ^b^	45.00 ± 0.33 ^a^	<0.01
RDW-CV (%)	19.20 ± 0.12	20.05 ± 0.17	20.80 ± 0.29	0.13
PDW (fL)	10.38 ± 0.06	11.75 ± 0.20	10.96 ± 0.17	0.16
MPV (fL)	9.58 ± 0.07	9.87 ± 0.08	9.56 ± 0.05	0.46
*p*-LCR (%)	22.25 ± 0.70	24.63 ± 0.81	21.76 ± 0.55	0.53
Plateletcrit (%)	0.69 ± 0.02	0.74 ± 0.01	0.80 ± 0.02	0.29
NRBC (10^9^/L)	0.15 ± 0.02	0.06 ± 0.01	0.09 ± 0.01	0.17
Neutrophils (10^9^/L)	3.77 ± 0.21	4.89 ± 0.43	4.64 ± 0.42	0.13
Lymphocytes (10^9^/L)	8.71 ± 0.25	8.50 ± 0.19	10.68 ± 0.57	0.21
Monocytes (10^9^/L)	0.92 ± 0.01	0.83 ± 0.03	1.17 ± 0.14	0.45
Eosinophils (10^9^/L)	0.20 ± 0.01 ^a^	0.14 ± 0.01 ^b^	0.12 ± 0.01 ^b^	0.05
Basophils (10^9^/L)	0.10 ± 0.01	0.07 ± 0.01	0.07 ± 0.01	0.35
Neutrophils (%)	27.22 ± 0.89	31.60 ± 1.50	27.66 ± 1.86	0.66
Lymphocytes (%)	63.62 ± 0.89	60.90 ± 1.34	64.64 ± 1.72	0.73
Monocytes (%)	6.97 ± 0.26	6.00 ± 0.22	6.54 ± 0.46	0.69
Eosinophils (%)	1.50 ± 0.08 ^a^	1.00 ± 0.07 ^ab^	0.74 ± 0.01 ^b^	0.03
Basophils (%)	0.70 ± 0.05	0.50 ± 0.07	0.42 ± 0.03	0.20
Biochemical indices				
AST (IU/L)	41.20 ± 4.43 ^a^	27.00 ± 2.42 ^b^	41.75 ± 3.90 ^a^	0.02
ALT (IU/L)	45.00 ± 3.65	41.50 ± 3.17	44.80 ± 4.65	0.76
ALP (IU/L)	425.67 ± 9.57	403.60 ± 5.72	399.60 ± 7.57	0.43
GGT (IU/L)	29.17 ± 1.34	31.00 ± 1.14	35.80 ± 1.60	0.37
TBIL (μmol/L)	1.02 ± 0.03 ^a^	1.10 ± 0.12 ^a^	0.72 ± 0.05 ^b^	0.02
DBIL (μmol/L)	0.37 ± 0.03	0.33 ± 0.05	0.24 ± 0.02	0.61

The data are represented as means ± SEM, *n* = 5. Different letters indicate significant differences (*p* < 0.05). CON, control; LPS, lipopolysaccharide; LPS + PAC, lipopolysaccharide + proanthocyanidin. WBC, White Blood Cell; RBC, Red Blood Cell; MCV, Mean Corpuscular Volume; MCH, Mean Corpuscular Hemoglobin Content; MCHC, Mean Corpuscular Hemoglobin Concentration; RDW-SD, Red Blood Cell Volume Distribution Width–Standard Deviation; RDW-CV, Red Blood Cell Volume Distribution Width–Coefficient Of Variation; PDW, Platelet Distribution Width; MPV, Mean Platelet Volume; *p*-LCR, Platelet Ratio; NRBC, Nucleated Red Blood Cell; AST, Alanine Transaminase; ALT, Aspartate Aminotransferase; ALP, Alkaline Phosphatase; GGT, Gamma-Glutamyltransferase; TBIL, Total Bilirubin; DBIL, Direct Bilirubin.

## Data Availability

Data are contained within the article.
